# Computational Study of Heat Transfer inside Different PCMs Enhanced by Al_2_O_3_ Nanoparticles in a Copper Heat Sink at High Heat Loads

**DOI:** 10.3390/nano10020284

**Published:** 2020-02-07

**Authors:** Nadezhda S. Bondareva, Nikita S. Gibanov, Mikhail A. Sheremet

**Affiliations:** Laboratory on Convective Heat and Mass Transfer, Tomsk State University, Tomsk 634050, Russia; bondarevans@mail.tsu.ru (N.S.B.); gibanov@mail.tsu.ru (N.S.G.)

**Keywords:** nanoparticles, phase change material, natural convection, high heat loads

## Abstract

The cooling of electronic elements is one of the most important problems in the development of architecture in electronic technology. One promising developing cooling method is heat sinks based on the phase change materials (PCMs) enhanced by nano-sized solid particles. In this paper, the influence of the PCM’s physical properties and the concentration of nanoparticles on heat and mass transfer inside a closed radiator with fins, in the presence of a source of constant volumetric heat generation, is analyzed. The conjugate problem of nano-enhanced phase change materials (NePCMs) melting is considered, taking into account natural convection in the melt under the impact of the external convective cooling. A two-dimensional problem is formulated in the non-primitive variables, such as stream function and vorticity. A single-phase nano-liquid model is employed to describe the transport within NePCMs.

## 1. Introduction

The advent of electronic technologies with increased power and expanding their scope is ongoing on continuously and a large role in the development of technology in recent decades has been played by heat removal systems. The requirements for a heat sink depend not only on capacities but they are also associated with geometric parameters. High heat dissipation in a small volume requires a high effective heat capacity of the system and intensive heat dissipation. In addition, it is often required that the entire system occupies a small volume. To increase performance, metal radiators are filled with phase change materials (PCMs), which not only allows for the extension of the operating time of the device. When the melting point is reached, a large amount of energy is spent on melting at a constant temperature, which allows the use of such materials for both thermal control and thermal energy storage systems [1,2,3,4]. In such systems, PCMs significantly reduce the operating temperature and make it possible to increase thermal electrical efficiency of a photovoltaic/thermal system [1,5,6,7,8]. Adding materials with a low melting point to building structures can reduce energy costs for air conditioning systems and reduce temperature fluctuations associated with daily thermal cycles [9,10,11,12].

To accelerate the heat transfer processes inside the PCM, various nanoadditives with high thermal conductivity can be used. For the present, there are a large number of scientific studies devoted to the effect of nanoparticles on the melting processes, which note both a positive effect on heat transfer and a negative one [13,14,15,16]. The effect of adding nanoparticles depends on many factors, including the thermal and geometric properties of the system, as well as the thermophysical properties of the materials [17].

In [15], thermal energy storage based on coconut oil with CuO nanoparitcles was analyzed. It was experimentally shown that melting occurs faster when nanoparticles are added. In [16], RT35HC enhanced by Al_2_O_3_ nanoadditives was used to lower the operating temperatures of the photovoltaic panel. It was shown that the use of a PCM heat sink can reduce the surface temperature by 34 °C at high heat fluxes, while the addition of nanoparticles to the PCM further reduces the temperature by 4.5 °C.

A numerical study of n-octadecane melting inside a closed copper radiator in the presence of a source with volumetric heat generation showed that the addition of nanoparticles can slightly reduce the temperature in the source at low concentrations (Φ = 1%), but at high nanoparticle concentrations, the melt circulation rate can be decreased by 57% (Φ ≥ 3%), which can lead to an increase in the temperature of the element [18].

In experimental study [13], the melting of n-octadecane with TiO_2_ nanoparticles (titanium oxide) inside the rectangular cavity heated from the side wall at a constant heat flux and thermally insulated on other sides was considered. The mass fraction of nanoparticles ranged from 0% to 4%. It was shown that at q” = 10,000 W/m^2^, the addition of 1, 2, and 4 wt % nanoparticles leads to a decrease in the quasi-steady Nusselt number by more than 10%, 36%, and 40%, respectively. This is related to the fact that natural convection plays a large role in heat transfer. However, an increase in viscosity leads to a decrease in the intensity of convective heat and mass transfer and an increase in thermal conductivity does not compensate for the effect of viscosity increasing. Heat transfer in the phase change materials is complicated by the presence of a moving border. Many studies have been shown that, at the initial stage, conductive heat transfer dominates in the region, however, with the growth of the melt region, an intensive regime of natural convection develops [18,19,20,21].

Nanoparticles have a complex effect on the processes of heat and mass transfer in liquids. On the one hand, there is an increase in the effective thermal conductivity of the suspension with increasing in conductive heat transfer. On the other hand, an increase in the melt viscosity affects hydrodynamics and can significantly reduce convective heat transfer. There are many numerical and experimental studies on the performance of heat sinks based on nano-enhanced phase change materials (NePCMs). In some of these studies, a positive effect of nanoparticles on heat transfer is noted, and, in others, there is a decrease in heat sink performance at high concentrations of nanoparticles. These two effects play a decisive role in the effectiveness of using nanoadditives. The intensity of thermal processes in a nano-enhanced PCM is determined not only by the parameters of the system and thermal conditions but also by the properties of base fluid and nano-sized particles. The melting temperature of the material is one of the determining factors in the heat transfer regimes for the system. This is evidenced by the studies devoted to the analysis of the influence of the PCM melting point on the efficiency of using heat sink [22,23]. Using the melting latent heat of materials allows for the absorption of a large amount of energy reaching the melting point.

Phase change materials with different melting points were used in experimental study [22]. The paraffin wax (*T_m_* = 56–58 °C, *L_m_* = 173.6 kJ/kg), n-eicosane (*T_m_* = 36.5 °C, *L_m_* = 237.4 kJ/kg), RT–54 (*T_m_* = 54 °C, *L_m_* = 200 kJ/kg), RT–44 (*T_m_* = 44 °C, *L_m_* = 250 kJ/kg), RT–35HC (*T_m_* = 35 °C, *L_m_* = 240 kJ/kg), and SP–31 (*T_m_* = 31 °C, *L_m_* = 210 kJ/kg) were employed for analysis. Heat transfer in a PCM-based pin-finned heat sink alumina radiator was studied under different heat loads. It was shown that at the highest considered power, the material with a high melting point RT–54 is turned out to be the most suitable. It should be also noted that at low critical temperatures (*T* = 45 °C) it is more efficient to use SP-31. At critical temperatures of *T_cr_* = 60 °C, paraffin wax with *T_f_* = 56–58 °C turned out to be the most effective one.

In [23], the performance of an alumina heat sink with two different PCMs, namely, n-eicosane and 1-hexadecanol, was studied. The authors showed that, to increase the productivity of the heat sink, the PCM with the highest melting point was preferable. It was also noted that, when using a PCM with a low *T_m_*, the heat sink performance increases at low operation temperature. Thus, the choice of PCM should be based on the operating conditions of the device, technical requirements, power, and other parameters. In [24], three different PCMs in finned heat sink were considered. The investigation showed an evolution of heat sink temperature for different PCMs. When the temperature of the phase transition is reached, the melting process begins, and the temperature, at the same time, reaches a constant level and practically does not change. The higher melting temperature of the material, the more the radiator heats up before the phase transition, accompanied by intense absorption of energy, begins. After the material has completely melted, the temperature in the region starts to rise again. Here, the latent heat of melting plays a large role, which affects the effective heat capacity of the heat sink and, therefore, the duration of the process.

In this paper, five different phase change materials with different melting points in the range from 28 to 81 °C with Al_2_O_3_ nanoparticles in a closed metal radiator to cool the source of constant volumetric heat release at high heat loads were considered. The two-dimensional problem of conjugated natural convection, taking into account phase transitions, was formulated in dimensionless variables such as the stream function, vorticity, and temperature with the smoothing of the jump in internal energy at the boundary of the phase transition. As a working liquid, the following PCMs were selected: n-octadecane, Capric acid, Lauric acid, RT–50, RT–80.

## 2. Mathematical Model

**Figure 1:**
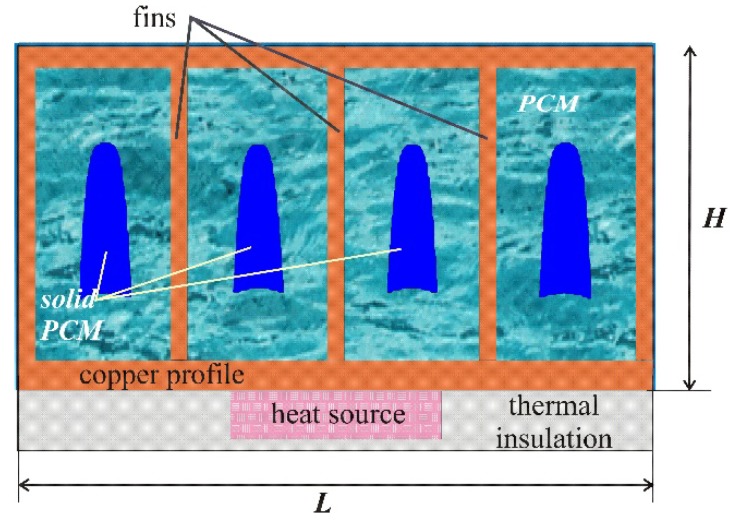
Domain of interest.

Figure 1 shows the model of the region under consideration, namely, a closed metal rectangular profile with dimensions of *L* × *H*, where *L* = 3.0 cm and *H* = 1.5 cm, and internal metal fins, between which the PCM enhanced by Al_2_O_3_ nanoparticles is located. A rectangular source of constant volumetric heat generation with the properties of silicon with a total power of 400 W per meter length is located under the metal profile.

The temperature of the «source–heat sink» system at the initial time coincided with the ambient temperature *T_0_* = 23 °C, which was considered constant. As the external boundary conditions, the following relations were set: the upper and side boundaries of the radiator were cooled by air convective cooling with a constant heat transfer coefficient *γ* = 20 W/(m^2^∙K), and the remaining boundaries were considered to be heat-insulated. Natural convection in a viscous melt has been described using the Boussinesq approximation. A nano-enhanced phase change material was considered as a single-phase medium with constant thermophysical properties.

The equations of natural thermogravitational convection in the melt have the following form [18]:

(1) $$\frac{\partial u}{\partial x}+\frac{\partial v}{\partial y}=0$$

(2) $${\left(\rho_{l}\right)}_{nm}\left(\frac{\partial u}{\partial t}+u\frac{\partial u}{\partial x}+v\frac{\partial u}{\partial y}\right)=-\frac{\partial p}{\partial x}+{\left(\mu_{l}\right)}_{nm}\left(\frac{\partial^{2}u}{\partial x^{2}}+\frac{\partial^{2}u}{\partial y^{2}}\right)$$

(3) $${\left(\rho_{l}\right)}_{nm}\left(\frac{\partial v}{\partial t}+u\frac{\partial v}{\partial x}+v\frac{\partial v}{\partial y}\right)=-\frac{\partial p}{\partial y}+{\left(\mu_{l}\right)}_{nm}\left(\frac{\partial^{2}v}{\partial x^{2}}+\frac{\partial^{2}v}{\partial y^{2}}\right)+{\left(\rho_{l}\beta_{l}\right)}_{nm}g\left(T-T_{m}\right)$$

The energy equations for an NePCM, taking into account the phase boundary, are written separately for solid and liquid phases [18]:

(4) $$\frac{\partial h}{\partial t}={\left(k_{s}\right)}_{nm}\left(\frac{\partial^{2}T}{\partial x^{2}}+\frac{\partial^{2}T}{\partial y^{2}}\right)$$

(5) $$\frac{\partial h}{\partial t}+u\frac{\partial h}{\partial x}+v\frac{\partial h}{\partial y}={\left(k_{l}\right)}_{nm}\left(\frac{\partial^{2}T}{\partial x^{2}}+\frac{\partial^{2}T}{\partial y^{2}}\right)$$

The interface motion satisfies the Stefan condition [k∂T∂n¯]=−LFVn. In the metal case with fins and the heater, the conduction equations with the source term *Q* were solved [18]:

(6) $$\frac{\partial T}{\partial t}=k_{1}\left(\frac{\partial^{2}T}{\partial x^{2}}+\frac{\partial^{2}T}{\partial y^{2}}\right)$$

(7) $${\left(\rho c\right)}_{2}\frac{\partial T}{\partial t}=k_{2}\left(\frac{\partial^{2}T}{\partial x^{2}}+\frac{\partial^{2}T}{\partial y^{2}}\right)+Q$$

Partial differential equations of natural convection together with the energy equations for the heat sink and heat source before the computational procedures were formulated in dimensionless non-primitive variables, such as stream function and vorticity. As the temperature scale Δ*T* = 60 °C was set so that the dimensionless temperature was determined as $\Theta =\left(T-T_{m}\right)/\Delta T$, where *T_m_* is the melting point of a PCM and as a length scale, the radiator height *L* = 1.5 cm was chosen, hence, X=x/L, Y=y/L. The value of the velocity scale was chosen $V_{0}=\sqrt{g\beta \Delta TL}$ and the dimensionless values of time, stream function, and vorticity were determined, respectively, from the following relations: $\tau =tV_{0}/L$, $\Psi =\psi /LV_{0}$, $\Omega =\omega L/V_{0}$.

As a result of transformations, the following equations were obtained for NePCM [18]:

(8) $$\frac{\partial^{2}\Psi }{\partial X^{2}}+\frac{\partial^{2}\Psi }{\partial Y^{2}}=-\Omega$$

(9) $$\frac{\partial \Omega }{\partial \tau }+U\frac{\partial \Omega }{\partial X}+V\frac{\partial \Omega }{\partial Y}=\frac{\mu_{nm}/\mu_{l}}{{\left(\rho_{l}\right)}_{nm}/\rho_{l}}\sqrt{\frac{Pr}{Ra}}\left(\frac{\partial^{2}\Omega }{\partial X^{2}}+\frac{\partial^{2}\Omega }{\partial Y^{2}}\right)+\frac{{\left(\rho_{l}\beta_{l}\right)}_{nm}/\left(\rho_{l}\beta_{l}\right)}{{\left(\rho_{l}\right)}_{nm}/\rho_{l}}\frac{\partial \Theta }{\partial X}$$

(10) $$\zeta \left(\varphi \right)\left[\frac{\partial \Theta }{\partial \tau }+U+V\frac{\partial \Theta }{\partial Y}\right]+\frac{\rho_{nm}L_{nm}}{\rho_{f}L_{f}}\cdot Ste\cdot \left[\frac{\partial \varphi }{\partial \tau }+U\frac{\partial \varphi }{\partial X}+V\frac{\partial \varphi }{\partial Y}\right]=\frac{\xi \left(\varphi \right)}{\sqrt{Ra\cdot Pr}}\left(\frac{\partial^{2}\Theta }{\partial X^{2}}+\frac{\partial^{2}\Theta }{\partial Y^{2}}\right).$$

In the energy equation, the smoothing function *φ*(*T*) is used, which determines the volume fraction of the melt and varies from 0 to 1 during the transition from solid material to melt, whereby a smooth transition of the enthalpy and thermophysical parameters of the material, expressed in functions *ζ*(*φ*) and *ξ*(*φ*), was determined:

(11) $$\varphi =\left\{ \begin{array}{ll} 0, & T<T_{m}-\eta \\ \frac{T-\left(T_{m}-\eta \right)}{2\eta }, & T_{m}-\eta \leq T\leq T_{m}+\eta \\ 1, & T>T_{m}+\eta \end{array}\right.$$

The energy equations, taking into account the volumetric heat generation for the source and profile, are as follows:

(12) $$\frac{\partial \Theta }{\partial \tau }=\frac{\alpha_{1}/\alpha_{0}}{\sqrt{Ra\cdot Pr}}\left(\frac{\partial^{2}\Theta }{\partial X^{2}}+\frac{\partial^{2}\Theta }{\partial Y^{2}}\right)$$

(13) $$\frac{\partial \Theta }{\partial \tau }=\frac{\alpha_{2}/\alpha_{0}}{\sqrt{Ra\cdot Pr}}\left(\frac{\partial^{2}\Theta }{\partial X^{2}}+\frac{\partial^{2}\Theta }{\partial Y^{2}}+Os\right)$$

The dimensionless equations contain the following dimensionless complexes, which are determined by the geometry parameters, thermal properties of the system, and the medium, as well as the properties of materials, which will be presented below: Rayleigh number $Ra=\frac{g\beta \Delta TL^{3}}{\nu \alpha_{l}}$, Prandtl number $Pr=\nu_{l}/\alpha_{l}$, Stefan number $Ste=L_{f}/\left(c_{l}\Delta T\right)$, Ostrogradsky number $Os=QL^{2}/\left(k_{2}\Delta T\right)$, and the Biot number Bi=γL/k.

The single energy equation was formulated for solid and liquid phases in a temperature formulation using the smoothing function *φ*, and it was solved without highlighting the melting front. Hydrodynamic equations were solved in a region with moving boundaries, which position at each time step was determined by isotherms obtained at this time step.

At the initial time *τ* = 0, the temperature in the entire system is coincided with the ambient temperature *Θ* = *Θ_out_*, and since the material was in the solid state, the values of the stream function and vorticity were equal to zero (*Ψ* = 0, *Ω* = 0). Boundary conditions in the dimensionless form for the presented statement of the conjugate problem are as follows:

- at the boundaries between system elements:
  at the border of the heat source: $k_{1}\frac{\partial \Theta_{1}}{\partial Y}=k_{2}\frac{\partial \Theta_{2}}{\partial Y}$;
  at the profile surface: $k_{0}\frac{\partial \Theta_{0}}{\partial Y}=k_{1}\frac{\partial \Theta_{1}}{\partial Y}$;
- at the outer borders:
  0≤ *X* ≤ 2, *Y* = 1: ${\frac{\partial \Theta }{\partial Y}|}_{M}=-Bi\left(\Theta_{M}-\Theta_{out}\right)$;
  *X* = 0 and *X* = 2, 0 ≤ *Y* ≤ 1: ${\frac{\partial \Theta }{\partial Y}|}_{0}=-Bi\left(\Theta_{0}-\Theta_{out}\right)$ and ${\frac{\partial \Theta }{\partial Y}|}_{N}=-Bi\left(\Theta_{N}-\Theta_{out}\right)$;
  at *X* = 0.6 and *X* = 1.4, –0.2 ≤ *Y* ≤ 0 and 0.6 ≤ *X* ≤ 1.4, *Y* = –0.2: $\frac{\partial \Theta }{\partial \bar{n}}=0$.

The dimensionless ambient temperature *Θ_out_* for each material was different, as it was determined by the melting temperature of the material and the temperature scale.

- The boundary conditions for the Poisson Equation (8) and vorticity Equation (9) *Ψ* = 0, $\Omega =-\nabla^{2}\Psi$ were applied for all solid boundaries of the melt region, including interphase. The transition conditions of the thermophysical properties of the material were expressed through a smoothing function:
  (14) $$\zeta \left(\varphi \right)=\frac{{\left(\rho_{s}c_{s}\right)}_{nm}}{\rho_{l}c_{l}}+\varphi \left(\frac{{\left(\rho c\right)}_{nm}}{\rho_{l}c_{l}}-\frac{{\left(\rho_{s}c_{s}\right)}_{nm}}{\rho_{l}c_{l}}\right)$$
  (15) $$\xi \left(\varphi \right)=\frac{{\left(k_{s}\right)}_{nm}}{k_{l}}+\varphi \left(\frac{k_{nm}}{k_{l}}-\frac{{\left(k_{s}\right)}_{nm}}{k_{l}}\right)$$

**Figure 2:**
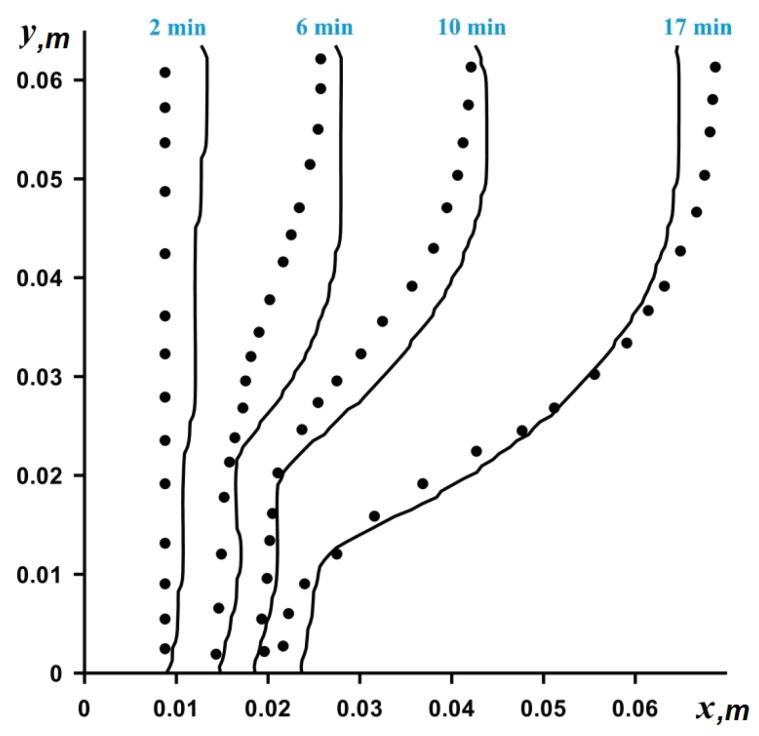
Comparison of obtained numerical data (solid line) and experimental data (dots) [29].

The numerical solution to Equations (8)–(13) was obtained on the basis of the finite difference method [25,26,27,28]. To discretize the convective terms in vorticity (Equation (9)) and energy (Equation (10)), we used the Samarskii monotonic difference scheme, and the diffusion terms in all equations were approximated based on the central differences with a second order of accuracy. The Poisson difference equation for the stream function was solved by the successive over-relaxation method. Samarskii local one-dimensional difference scheme for the approximation of the vorticity and energy equations was applied. Using this described method, the developed computational code was verified, employing the experimental data for the gallium melting. A numerical algorithm was applied in solving the problem of gallium melting in a rectangular region and showed good agreement with experimental results [29] (see Figure 2).

It should be noted that for the phase change problems, the presence of a moving boundary requires the use of a more detailed mesh. In the present study, a grid with 481 × 201 nodes was taken. Solving melting problems are time-consuming, due to the fact that the melting and solidification processes occur much more slowly than hydrodynamic processes. Therefore, problems associated with phase transitions are solved, as a rule, in the two-dimensional approximation. Moreover, using the non-primitive variables “stream function and vorticity” with the finite difference method allows for a reduction in the computational time due to both a smaller number of governing equations compared with the primitive variables “velocity and pressure” and an absence of the global iterations that are used, e.g., in the finite volume method with SIMPLE-like algorithms.

As for the considered two-dimensional problem, it is assumed that the third size is greater than *L*, and such an approach characterizes an opportunity to analyze 2D problem because the heat transfer rate will be similar to the 3D data. A comparison between 2D and 3D models for convective heat transfer has been performed earlier in [30,31,32,33].

Thus, the results of 2D and 3D models for magnetohydrodynamic (MHD) natural convection in a cavity were compared in [31]. The transverse elongation in the parallelepiped was varied from 0.2 to 5. A comparison of the obtained data for different models showed that the influence of the side walls decreases with the lengthening of the cavity and, as a result, 3D laminar natural convection can be replaced by 2D data for the heat transfer rate and fluid flow in the central part of the cavity. A study of the melting of n-octadecane in the cubic cavity (see [33]) showed that the isotherms in the middle cross section of the cube slightly differ from the two-dimensional model, while neglecting differences can be found in the flow structures for 2D and 3D models.

Therefore, the present analysis can be performed using the two-dimensional approximation.

## 3. Thermophysical Properties of the System Components

The system under consideration consisted of a volumetric heat generation source with silicon properties combined with the closed copper radiator with copper finning. As the nanoadditives the Al_2_O_3_ particles with diameter *d* = 59 × 10^−9^ m were chosen, the properties of these system components are presented in Table 1.

Five different PCMs were considered in the present study in the range of melting temperatures from 28 to 81 °C. The considered PCM properties are presented in Table 2.

As a model for describing nanoparticles suspended in a phase change material, a single-phase model, which assumes a uniform particle distribution in the volume, was applied. The properties of NePCM were considered constant within one phase. The density of NePCM was determined by the densities of the corresponding components and the mass concentration Φ of nanoparticles:

$$\begin{array}{l} {\left(\rho_{l}\right)}_{nm}=\left(1-\Phi \right)\rho_{l}+\Phi \rho_{np}\\ {\left(\rho_{s}\right)}_{nm}=\left(1-\Phi \right)\rho_{s}+\Phi \rho_{np} \end{array}$$

In turn, the volumetric heat capacity and the coefficient of thermal volume expansion were determined similarly:

$$\begin{array}{l} {\left(\rho_{l}c_{l}\right)}_{nm}=\left(1-\Phi \right)\left(\rho_{l}c_{l}\right)+\Phi \left(\rho_{np}c_{np}\right)\\ {\left(\rho_{s}c_{s}\right)}_{nm}=\left(1-\Phi \right)\left(\rho_{s}c_{s}\right)+\Phi \left(\rho_{np}c_{np}\right) \end{array}$$

In this study, the thermal conductivity model [39] for nanofluids, which takes into account the influence of particle size, concentration, and base fluid properties, was used. The authors proposed a correlation in which the effective thermal conductivity is the following sum: $k_{eff}=k_{static}+k_{Brownian}$ where *k_static_* has been found from the Maxwell equation, and the additional term *k_Brownian_* corresponds to the Brownian motion of particles:

$${\left(k_{l}\right)}_{nm}=k_{l}\frac{k_{np}+2k_{l}-2\left(k_{l}-k_{np}\right)\Phi }{k_{np}+2k_{l}+\left(k_{l}-k_{np}\right)\Phi }+5\cdot 10^{4}\beta_{\lambda }\Phi \rho_{l}c_{l}\sqrt{\frac{\kappa T}{\rho_{np}d_{np}}}f\left(T,\Phi \right)$$

where the Brownian motion of the molecules in the melt represented as the second term was taken into account, the thermal conductivity in a solid material was respectively determined from the Maxwell’s relation:

$$k_{nm}=k_{l}\frac{k_{np}+2k_{l}-2\left(k_{l}-k_{np}\right)\Phi }{k_{np}+2k_{l}+\left(k_{l}-k_{np}\right)\Phi }$$

Here, $\kappa =1.381\cdot 10^{-23}J/K$ is the Boltzmann constant. In [40], the new empirical correlations for β and *f*(*T*,Φ) were obtained experimentally considering nanoparticles of Al_2_O_3_, ZnO, and CuO. Correlations $\beta_{\lambda }=8.4407{\left(100\Phi \right)}^{-1.07304}$ and the function *f*(*T*, Φ):

$$f\left(T,\Phi \right)=\left(2.817\cdot 10^{-2}\Phi +3.917\cdot 10^{-3}\right)\frac{T}{T_{0}}+\left(-3.0669\cdot 10^{-2}\Phi -3.91123\cdot 10^{-3}\right),\;T_{0}\;=\;273\;{}^{\circ}K$$

were obtained for nanoparticles of Al_2_O_3_ for the following concentration range: 1% ≤ Φ ≤ 10%.

The melt viscosity was determined as [41]: $\mu_{nm}=0.983\cdot e^{12.959\Phi }\mu_{f}$.

The latent heat decreased with the increasing concentration of nanoparticles:$L_{nm}=\frac{\left(1-\Phi \right)\rho_{f}L_{f}}{\rho_{nm}}$.

## 4. Results and Discussion

**Figure 3:**
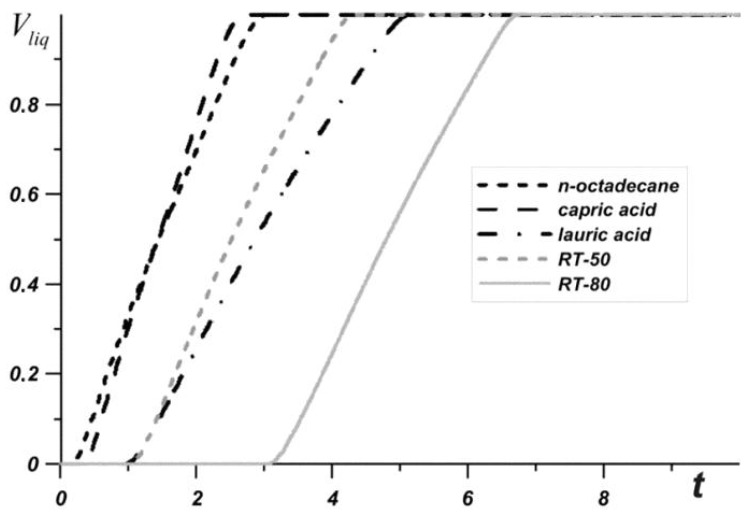
Liquid volume fraction versus time.

Figure 3 shows the average source temperature versus time for five different PCMs: n-octadecane with a melting point of 28.05 °C, capric acid with a melting point of 32 °C, lauric acid with a melting point of 46 °C, RT–50 with a melting point of 49 °C, and RT–80 with a melting point of 81 °C for various nanoparticles loadings. In each case, it can be seen that the temperature increases evenly, while the temperature graph has two inflections corresponding to the beginning of the melting of the material and the end of the melting process. Exceeding the melting temperature is accompanied by the intense absorption of energy, which causes the temperature to rise more slowly.

The phase change material with the lowest melting point begins to melt first, and during the entire melting process, the temperature in the source is lower when using an n-octadecane-based heat sink compared to other materials. However, this material melts very quickly and the temperature of paraffin rises rapidly. However, the source heats to 60 °C longer in the case of the n-octadecane-based heat sink. The remaining materials begin to melt later. It is worth noting that for n-octadecane, capric acid, and RT–50, the temperature in the source differs by no more than 6 degrees. Capric acid and RT–50 have low latent heat and therefore melt faster than n-octadecane and lauric acid, respectively.

**Figure 4:**
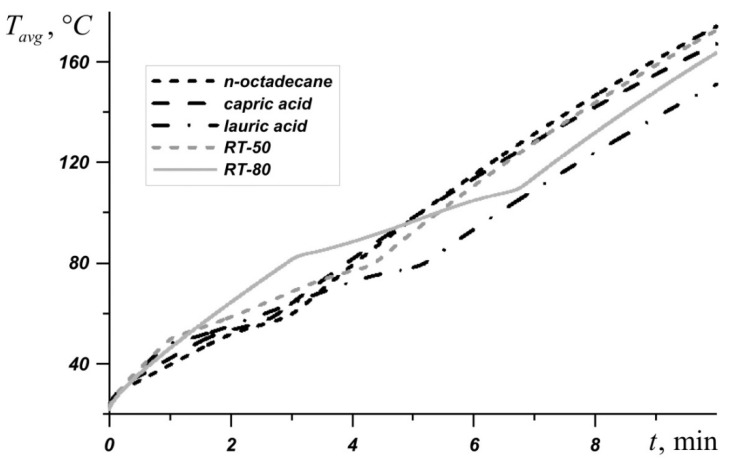
Average source temperature for five different phase change materials (PCMs).

Later than the others, RT–80 begins to melt, so for some time the temperature in the source is higher than in other cases (see Figure 4), however, by the end of the melting process, the temperature difference in the source reaches 8–11 degrees compared to n-octadecane, capric acid and RT–50. The heat source cooled by the lauric acid reaches a temperature of 80 °C later than other considered materials. It is worth noting that after four minutes of heating, the lowest temperature is observed in the heat sink based on the lauric acid. This PCM has the smallest one of the largest latent heat values and a sufficiently high heat capacity both in the solid state and in the melt.

**Figure 5:**
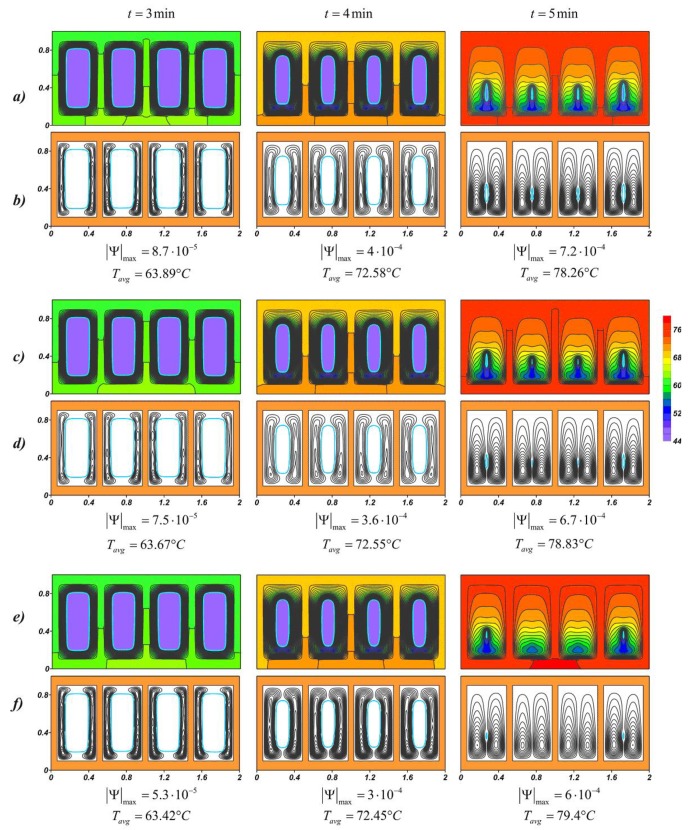
Temperature fields and streamlines for lauric acid at different time moments: (**a**,**b**) *Φ* = 2%; (**c**,**d**) *Φ* = 4%; (**e**,**f**) *Φ* = 6%.

Natural convection develops in the liquid material due to the temperature difference between the inner surface of the profile and the movable boundary of the yet-molten material. Figure 5 shows the temperature fields and streamlines for lauric acid at various nanoparticle loadings, and for each case, the maximum value of the stream function and the average source temperature at a given time are presented. At the initial time intervals around each solid region, two narrow convective cells develop along the vertical walls. The closed form of the radiator contributes to the rapid heat dissipation inside the highly heat-conducting casing; therefore, the heat spreads evenly around the perimeter. The maximum values of the stream function decrease with an increase in the concentration of nanoparticles, however, the melt region expands faster, due to the fact that thermal conductivity prevails at this stage. The intensification of conductive heat transfer leads to a slight decrease in the source temperature, this effect is weak and the temperature at time *t* = 3 min differs by only 0.47 °C for 2% and 6%.

**Figure 6:**
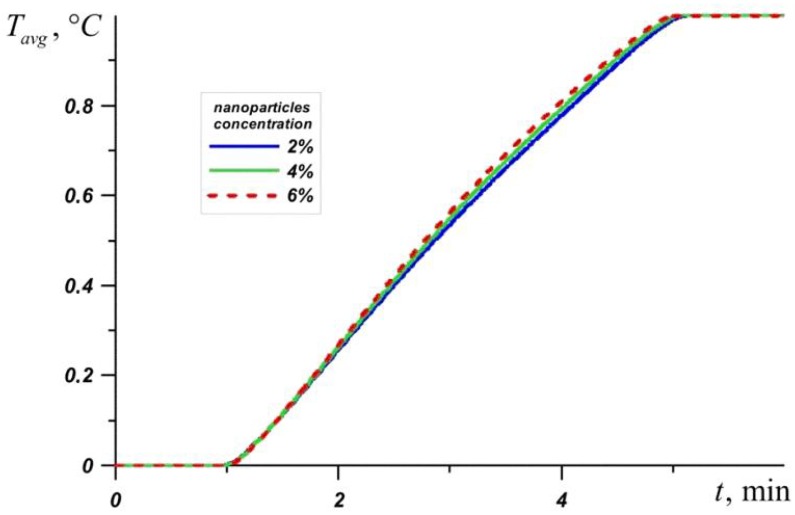
Liquid volume fraction versus time for lauric acid.

With the development of natural convection and a decrease in the solid region, the heated melt of lauric acid accumulates in the upper part. A cold downward flow descends to the lower slab, where the material is melted later and the cores of the circulation cells are located below. At the time *t* = 5 min, when the material almost melted, the opposite effect is observed, namely, the higher mass fraction of nanoparticles, the higher average temperature of the source. The expansion of the melt region and a constant increase in the temperature of the metal surface lead to the intensification of mass transfer, which can be seen from the maximum values of the stream function ${\left|\Psi \right|}_{\max}$. The effect of natural convection on temperature fields begins to prevail in NePCM. In this way, an increase in the melt viscosity due to the addition of nanoadditives critically affects the cooling process. In addition, the presence of nanoparticles in the material reduces its latent heat, which also accelerates the melting process (see Figure 6) and reduces the overall effective heat capacity of the system after 5 min from the start of source operating.

**Figure 7:**
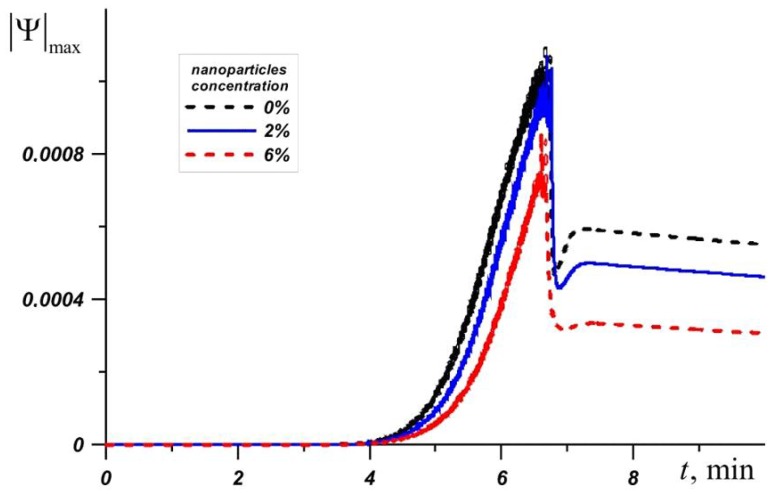
Maximum value of the stream function for RT-80 at different nanoparticle concentrations.

RT–80 has the highest melting point of all the phase change materials under consideration, so the temperature of the source quickly reaches 80 °C in this case. Figure 7 shows the change in the maximum value of the stream function at Φ = 0%, Φ = 2%, and Φ = 6%. Before the four minutes from the moment the source is turned on, the system heats up to the melting temperature of RT–80. This is followed by a monotonic increase in ${\left|\Psi \right|}_{\max}$ and the influence of nanoparticles increases over time. The addition of nano-sized particles of Φ = 6% reduces ${\left|\Psi \right|}_{\max}$ by about 1.5 times or more.

**Figure 8:**
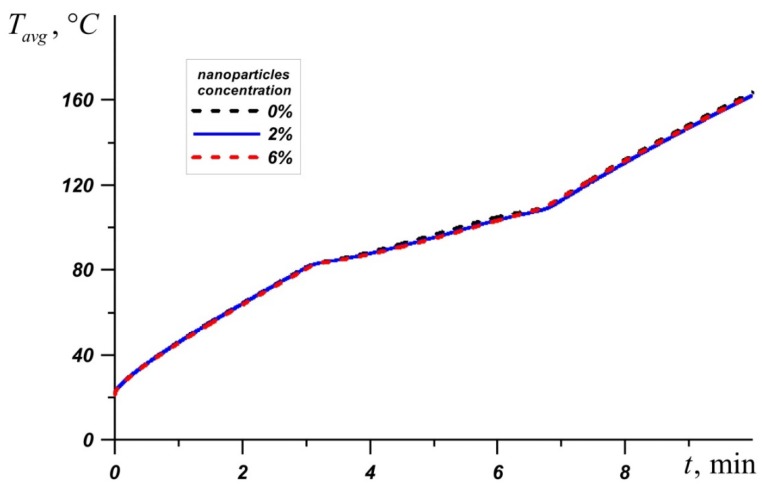
Average source temperature for RT-80 at different nanoparticle concentrations.

It should be noted that the addition of nanoparticles to the material does not lead to a significant decrease in the heater temperature. In the cases under consideration, at specific points in time, the temperature in the source decreases by no more than 1.5 °C with an increase in the concentration of nanoparticles, which is a relatively small difference in the considered thermal conditions. A high concentration of nanoparticles significantly reduces the intensity of the circulation of the melt, which leads to a decrease in the intensity of heat transfer. At the same time, the material melts faster due to a decrease in latent melting energy. Thus, an increase in the efficiency of the heat sink is observed only at a 2% concentration. At higher concentrations, the heater temperature decreases slightly, or vice versa, which leads to a decrease in heat transfer between the source and the heat sink (Figure 8).

**Figure 9:**
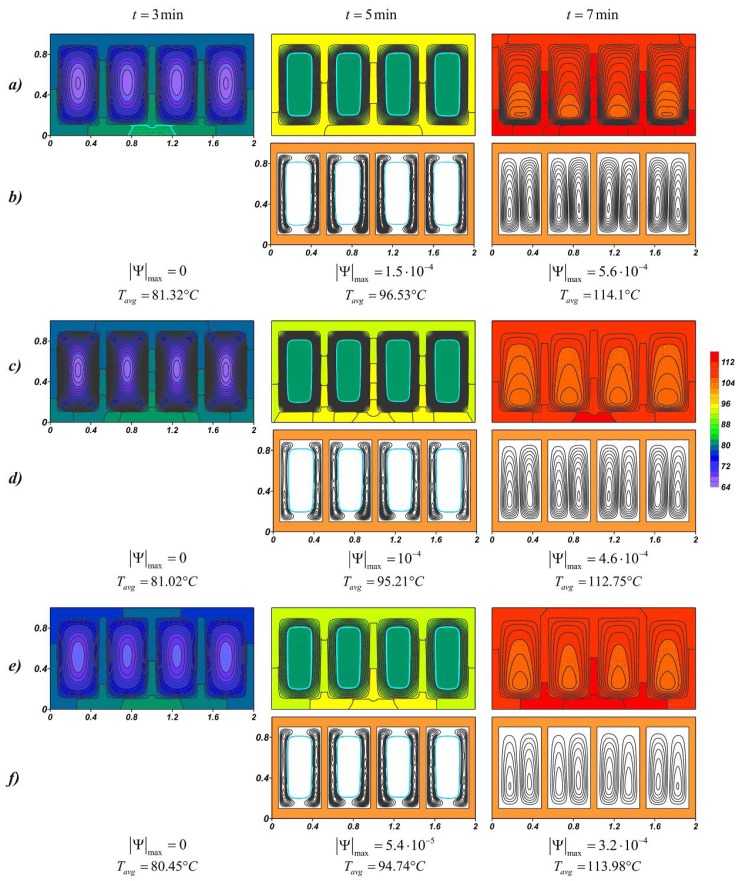
Temperature fields and streamlines for RT-80 at different time moments: (**a**,**b**) *Φ* = 0%; (**c**,**d**) *Φ* = 2%; (**e**,**f**) *Φ* = 6%.

The temperature fields shown in Figure 9 at the time *t* = 3 min present wide-spaced isotherms in the radiator and more densely located isotherms in the material on the inner surface of the profile, indicating a faster heat distribution in the radiator compared to the nano-enhanced material. When heating solid paraffin wax, before the phase transition, the temperature difference of the source for pure paraffin and paraffin with nanoparticle concentrations of 6% reaches 0.87 °C. When the radiator temperature exceeds 81 °C and the melting process begins, the effect of adding the nanoparticles increases, namely, the temperature difference for pure material and NePCM at Φ = 6% increases to 1.79 °C. After the melt volume reaches 100%, the average temperature of the source grows more rapidly. Due to convective heat and mass transfer, the lowest temperatures are observed in the lower part of the melt region. Moreover, in the case of Φ = 2%, the isotherms in the melt are less condensed to the walls, which indicate a more intense conductive heat transfer, as a result of which the observed *T_avg_* is lower by 1.35 °C at time *t* = 7 min and at higher mass particle concentrations. An increase in viscosity strongly affects the thermal processes in the system, namely, a 43% decrease in ${\left|\Psi \right|}_{\max}$ is accompanied by an increase in the average temperature of the source. The average heater temperature at Φ = 6% was only 0.12 °C lower than in the case of pure paraffin and at the same time by 1.23 °C higher than at Φ = 2%. Thus, paraffin with 2 wt % nanoparticles shows the best results. With the disappearance of the unmelted material, the maximum values of the stream function sharply decrease up to two times.

**Figure 10:**
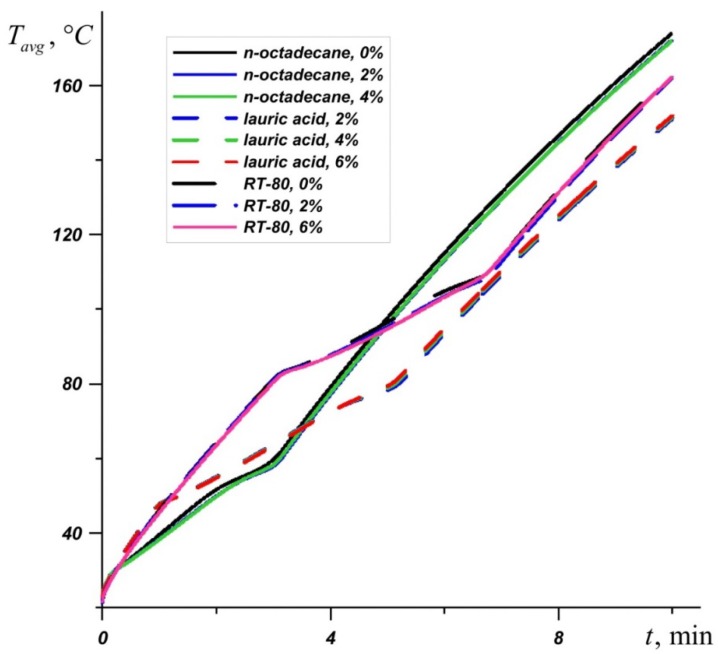
Evolution of average temperature in the source for n-octadecane, lauric acid, and RT-80 for different percentages of nanoparticles.

An inclusion of nanoparticles to the material leads to an increase in the melt viscosity and an improvement of conductive heat transfer. Figure 10 shows a graph of the average source temperature for three different PCMs at different concentrations of nanoadditives. At different stages of heating and different PCMs, nanoparticles give different effects for heat transfer processes. It can be seen that the most effective concentration of Al_2_O_3_ is Φ = 2%, which is most noticeable on the temperature graph *T_avg_* for n-octadecane. For RT–80, at the initial stages and during heating until the material is completely melted, the lowest temperatures are observed at a high concentration of nanoparticles Φ = 6%. However, after the melt volume reaches 100%, the PCM with 2% particle addition is most effective.

## 5. Conclusions

A numerical study of the effect of the Al_2_O_3_ nanoparticles on heat and mass transfer inside a closed system consisting of a volumetric heat generation element and an NePCM-based heat sink at high heat loads was carried out. The thermal characteristics of the system, including the position of the interface, the melt volume, the average temperature in the source, and the temperature distributions at fixed time points for five different materials (n-octadecane, Capric acid, Lauric acid, RT-50, and RT-80) were obtained. An analysis of the results showed that n-octadecane, lauric acid, and RT-80 are the most effective for cooling at high heat generations. Since n-octadecane begins to melt earlier than the other materials reviewed, the average temperature in the source at the initial minutes of heating is less than in other cases. However, the PCM with a melting point of 28.05 °C melts rather quickly, after which the heating element rapidly heats up. At critical temperatures above 70 °C, in the framework of the problem under consideration, lauric acid with a melting point of 46 °C is the most suitable phase change material, since after four minutes of melting of this material, the lowest temperatures are observed in the heat source.

An addition of nanoparticles has a small effect on the productivity of the heat sink and can reduce the temperature in the source by only 1.5 °C, however, for the five materials considered, depending on the heat and mass transfer conditions in the system, it can have both positive and negative effects. The most effective concentration, with which the ratio of the effects of increasing thermal conductivity and increasing viscosity was the most suitable is Φ = 2%. An increase in the mass fraction of particles from 2% to 6% leads to a significant weakening of the convective circulation of the melt and can lead to a decrease in the critical operating time.

## Figures and Tables

**Table 1:** Properties of nanoparticles and other materials.

Material	*k*, W/(m∙K)	*c*, J/(kg∙K)	*ρ*, kg/m^3^	*β*, K^−1^
Aluminum oxide (nanoparticles) (*d* = 59 × 10^−9^ m) [34]	36	765	3600	7.8 × 10^−6^
Copper (radiator)	401	385	8900	–
Silicon (heat source)	148	714	2330	–

**Table 2:** Properties phase change materials.

Phase Change Material	*T_m_*, °C	*L_f_*, kJ/kg	*k_s_/k_l_*, W/(m∙K)	*ρ_s_/ρ_l_*, kg/m^3^	*c_s_/c_l_*, J/(kg∙K)	*μ*, Pa∙s	*β*, K^−1^
n-octadecane [35]	28.05	241	0.39/ 0.157	814/ 770	1900/ 2200	3.8 × 10^−3^	8.5 × 10^−4^
Capric acid [36]	32	152.7	0.372/0.153	1018/888	1900/2400	2.7 × 10^−3^	10^−3^
Lauric acid [37]	46	187.2	0.16/0.14	940/885	2180/2390	8 × 10^−3^	8 × 10^−4^
RT–50 [38]	49	168	0.2	780	2000	4.8 × 10^−3^	6 × 10^−4^
RT–80 [34]	81	175	0.2	920/770	2400/1800	7.2 × 10^−3^	10^−3^

## Nomenclature

*Bi*	the Biot number
*c*	specific heat, J K^−1^ kg^−1^
*d*	diameter, m
*g*	gravitational acceleration, m s^−2^
*H*	cavity height, m
*h*	specific enthalpy, J kg^−1^
*k*	thermal conductivity, W K^−1^ m^−1^
*L*	cavity length, m
*L_m_*	fusion energy or latent heat of melting, J kg^−1^
*Os*	Ostrogradsky number
*p*	pressure, Pa
*Pr*	Prandtl number
*Q*	heat transfer rate per unit of volume, W m^−3^
*Ra*	Rayleigh number
*Ste*	Stefan number
*t*	time, s
*T*	temperature, K
*T_m_*	melting temperature
*u*, *v*	velocity components in Cartesian coordinate along *x* and *y*, m s^−1^
*U*, *V*	dimensionless velocity components
*x*, *y*	Cartesian coordinate, m
*X*, *Y*	dimensionless Cartesian coordinates
**Greek Symbols**	
*α*	thermal diffusivity, m^2^ s^−1^
*β*	coefficient of thermal expansion, K^−1^
*γ*	heat transfer coefficient, WK^−1^m^−2^
*η*	smoothing parameter (or melting temperature range), K
Θ	dimensionless temperature
*μ*	dynamic viscosity, Pa s
*ν*	kinematic viscosity, m^2^ s^−1^
*ρ*	density, kgm^−3^
*τ*	dimensionless time
Φ	nanoparticles volume fraction
*φ*	volume fraction of the melt
*ψ*	stream function, m^2^ s^−1^
Ψ	dimensionless stream function
*ω*	vorticity, s^−1^
Ω	dimensionless vorticity
**Subscripts**	
0	initial condition or ambient
1	radiator
2	heater
*l*	liquid
*m*	melting
*nm*	nanomaterial
*np*	nanoparticle
*s*	solid
